# Specific B- and T-cell populations are associated with cognition in patients with epilepsy and antibody positive and negative suspected limbic encephalitis

**DOI:** 10.1007/s00415-020-10158-1

**Published:** 2020-08-20

**Authors:** Christoph Helmstaedter, Niels Hansen, Pitshaporn Leelaarporn, Kerstin Schwing, Demet Oender, Guido Widman, Attila Racz, Rainer Surges, Albert Becker, Juri-Alexander Witt

**Affiliations:** 1 Department of Epileptology, University Hospital Bonn, Building 83 Venusberg-Campus 1, 53127 Bonn, Germany; 2 Department of Neuropathology, University Hospital, Bonn, Germany; 3 SEIN Epilepsy Center, Hemsteede, The Netherlands; 4 Department of Psychiatry and Psychotherapy, University Medical Center Göttingen, Von-Siebold-Str. 5, 37075 Göttingen, Germany

**Keywords:** Epilepsy, Limbic encephalitis, Antibodies, T-cells, B-cells, Cognition

## Abstract

**Objective:**

Neuropsychological impairments are major symptoms of autoimmune limbic encephalitis (LE) epilepsy patients. In LE epilepsy patients with an autoimmune response against intracellular antigens as well as in antibody-negative patients, the antibody findings and magnetic resonance imaging pathology correspond poorly to the clinical features. Here, we evaluated whether T- and B-cells are linked to cognitive impairment in these groups.

**Methods:**

In this cross-sectional, observational, case–controlled study, we evaluated 106 patients with adult-onset epilepsies with a suspected autoimmune etiology. We assessed verbal and visual memory, executive function, and mood in relation to the presence or absence of known auto-antibodies, and regarding T- and B-cell activity as indicated by flow cytometry (fluorescence-activated cell sorting = FACS, peripheral blood = PB and cerebrospinal fluid = CSF).

**Results:**

56% of the patients were antibody-negative. In the other patients, auto-antibodies were directed against intracellular antigens (GAD65, paraneoplastic: 38%), or cellular surface antigens (LGI1/CASPR2/NMDA-R: 6%). Excluding LGI1/CASPR2/NMDA-R, the groups with and without antibodies did not differ in disease features, cognition, or mood. CD4+ T-cells and CD8+ T-cells in blood and CD4+ T-cells in CSF were prominent in the auto-antibody positive group. Regression analyses indicated the role education, drug load, amygdala and/or hippocampal pathology, and CD4+ T-cells play in verbal memory and executive function. Depressed mood revealed no relation to flow cytometry results.

**Conclusion:**

Our results indicate a link between T- and B-cell activity and cognition in epilepsy patients with suspected limbic encephalitis, thus suggesting that flow cytometry results can provide an understanding of cognitive impairment in LE patients with autoantibodies against intracellular antigens.

## Introduction

Autoimmune encephalitis (AE) is associated with structural temporal lobe abnormalities, epileptic seizures, dynamic and sometimes progressive memory impairment and psychiatric disturbances, including mood dysfunction or psychosis [43]. Early diagnosis of AE relies on the clinical syndrome and more specific diagnostic results, including the detection of autoantibodies, which help in further classifying the disease [14].

Recent diagnostic criteria of AE include the bilateral involvement of mesiotemporal structures found on magnetic resonance imaging (MRI) of the brain [14]. However, clinical experience suggests that AE patients can also present with asymmetric or unilateral abnormalities in the limbic structures when examined via T2 MRI or fluid attenuated inversion recovery (FLAIR) MRI [43, 47], or even with normal limbic structures when scanned with MRI [8]. In MRI-positive patients, the brain’s limbic regions and especially the amygdalae and/or the hippocampi can appear inflamed and swollen, but tend to resolve later on [49]. Additionally, distinct white matter changes are sometimes observed [44, 48]. The course of limbic encephalitis (LE) can ultimately end in irreversible hippocampal damage and related memory impairment [9, 11, 19, 35]. Memory performance of LE patients with auto-antibodies against voltage gated potassium channels (VGKC) has been demonstrated to correlate with antibody load [5] and hippocampal damage in terms of CA3 cell loss in LGI1 patients [35]. The latter finding resembles that reported in regard to the relation of hippocampal cell loss and memory in chronic mesial temporal lobe epilepsies [53]. Early and successful immunological treatment and tumor resection (as in paraneoplastic LE) can reverse and improve or stabilize neurocognitive and behavioral problems [27].

Biomarkers (i.e., MRI, antibodies, seizures, cognitive and psychiatric impairment) are most important for diagnosing the disease early on, monitoring its progression, and then treating it successfully. Overall, this is better applied to those patients with LE with auto-antibodies against cell-surface antigens [e.g., LGI1 (Leucin rich glioma inactivated 1), CASPR 2 (Contactin-associated protein-like 2) or NMDA-R (*N*-methyl-d-aspartate receptor)] rather than those with intracellular antigens [e.g., Hu, Ma2, and less frequently CV2 (cronveinten 2)/CRMP5 (collapsing response mediator protein 5) and amphiphysin] [43]. In this regard, one major challenge is the patient group with new-onset epilepsies. Their clinical features suggest the presence of LE, but the data fail to match all the criteria for definitively diagnosing LE [14], especially those patients who are negative for the panel of known antibodies [45].

Flow cytometry (fluorescence-activated cell sorting analysis) is an advanced method suitable for use in the CSF or PB in autoimmune brain diseases such as multiple sclerosis (MS), and it might also be well-suited for unraveling the immune mechanisms underlying a suspected LE [13, 25, 26]. Based on the evidence gathered from the preliminary findings first obtained from a few GAD65 antibody-positive patients [50, 51] and then later on in a larger series [16, 17], we hypothesized that T- and B-lymphocytes could serve as additional potential biomarkers in LE, and thereby help clarify the variance in otherwise unexplained neurocognitive symptoms. However, when examining the cognitive and behavioral problems in epilepsy, many other etiological factors must be taken into consideration such as the active epilepsy, the drug treatment, the static and sometimes progressive morphological structural lesions, plus other patient variables [24].

Using a multifactorial approach, we evaluated the relative contribution of demographics, clinical data, structural MRI findings, and immunological data to determine the degree of cognitive impairment for a group of 124 patients with suspected LE. When taking into account all the available data, our working hypothesis suggests that a relationship exists between the amount of immune cells in CSF and PB as determined by flow cytometry analyses and the cognitive performance of LE patients.

## Methods

### Subjects

At the Department of Epileptology, University Hospital Bonn, Germany, between 2014 and 2016, 124 patients were examined for suspected autoimmune LE due to the presence of late-onset subacute epilepsies, with cognitive and/or psychiatric symptoms, and also because other reasonable alternative causes could be excluded [14].

All patients had peripheral blood (PB) and cerebrospinal fluid (CSF) extracted for autoantibodies identification and immune cells count. From the group of 124 patients, 18 patients were excluded from the analysis due to a lack of neuropsychological evaluations. In total, our study was comprised of 106 LE patients who had undergone neuropsychological evaluation at the time when CSF and PB were extracted and an MRI was performed. Demographic information (age, sex, and education), neuropsychological and clinical data (i.e., epilepsy onset, duration of epilepsy, the number of antiepileptic drugs taken, the number of psychoactive drugs, including antidepressants and neuroleptics, and structural MRI volumetric data) were collected retrospectively from medical files and records located in the databases of the Department of Epileptology, University Hospital Bonn.

This work was carried out in accordance with The Code of Ethics of the World Medical Association (Declaration of Helsinki) for experiments involving humans. Written informed consent was obtained from all patients and the study was approved by our local Ethics Committee (# 222/16).

### Neuroimaging data acquisition

To examine the relationship between morphological structural changes in the brain and cognition and mood, brain scans were obtained utilizing a 3-T MRI scanner at the Department of Neuroradiology (Philips Medical Systems, Germany) or the Life&Brain Institute (Magnetom Trio, Siemens, Germany). The structural MRI findings were categorized as normal, swollen, hyperintense, or atrophied hippocampi and/or amygdalae. The abnormalities in the hippocampus and/or amygdala were categorized as unilateral or bilateral. Three procedures were available for categorizing the MRI data: a visual inspection by neuroradiologists; an automated volumetry based on Freesurfer as described by Wagner et al. in 2015 [49]; and MAP/07, and a morphometric analysis program developed by J. Huppertz [6].

### Immunophenotyping and auto-antibody identification

Immune cells and their subsets were isolated and identified using the flow cytometry technique. The two main immune cells analyzed in this study were T-lymphocytes (T-cells) and B-lymphocytes (B-cells) which were categorized according to their cluster of differentiation (CD) and MHC class II, cellular surface receptors, into human leukocyte antigen DR isotype (HLA-DR+) CD4+ T-cells, HLA-DR+  CD8+ T-cells, and CD19+ B-cells from both CSF and PB. We utilized the fluorescences HLA-DR-ECD, CD4-APC, 700CD8-PacificBlue, and CD19-APC-Alexafluor700. The number of cells was counted per milliliter (cells/ml). We obtained at least 3 ml of CSF per patient. We measured the cell amounts and percentages of T-cells per total leucocytes on a BD LSR Fortessa flow cytometer (BD Bioscience, California, USA) and the data were analyzed with Kaluza software (Beckman Coulter GmbH, Life Science, Krefeld, Germany) and the following fluorochrome-conjugated antibodies (Beckman–Coulter). The gating strategy for identifying PB and CSF leukocytes subsets was performed via mainstream, cell line lineage markers. We focused on T- and B-cell populations which, according to previous investigations, pointed towards a relevant impact of these cell populations on limbic encephalitis [13]

The results of the auto-antibody testing were categorized into three subgroups: (1) antibodies against intracellular targets (IAB+); (2) antibodies against membrane surface antigens (SAB+); and (3) no detection of specific antibodies (AB−). Commercial kits were used for diagnostic procedures including: Amphiphysin, CV2, PNMA2 [Ma2/Ta; paraneoplastic antigen Ma2), Ri, Sox1, Yo, Zic4 and GAD65 (Glutamate acid decarboxylase 65] using semiquantitative immunoblots according to the manufacturer’s guidelines (EUROLINE PNS 12, Euroimmun, DL 1111-1601-7 G) coated with recombinant antigen or antigen fragments with serum diluted 1:100 and CSF 1:1. Complementary, indirect immunofluorescence was applied relying on HEK293-cells (Human Embryonic Kidney 293) with an overexpression of the individual antigens on the cell-surface (IIFT: ‘Autoimmune-Enzephalitis-Mosaik1’, Euroimmun, FA 1120-1005-1; GAD65-IIFT, Euroimmun, FA 1022-1005-50), including NMDAR-, CASPR-, LGI1- and GAD65-autoABs (dilution: serum 1:10; CSF 1:1) following the manufacturer’s protocol.

### Neuropsychological assessments

Neuropsychological memory tests were conducted on all patients to diagnose LE, determine a phenotype, and then obtain a baseline assessment for eventual follow-up examinations. While all patients underwent memory testing, 89 underwent additional assessment for attention and executive function, and 93 were assessed for mood.

To evaluate verbal memory, we the “Verbaler Lern- und Merkfähigkeitstest VLMT” [21, 32] which is a word list learning task, and is the German adaptation of the Rey Auditory Verbal Learning test (RAVLT). Our parameters of interest included learning over five learning trials, delayed free recall performance (absolute), loss of learned words over time, and recognition (minus errors).

To assess visual memory capacity, we employed the “Diagnosticum für Cerebralschädigung-Revised/DCS-R”, a design list learning task [22, 30]. We analyzed learning over five trials, performance in the last learning trial, and delayed recognition (minus errors).

Both tests, either taken alone or and in combination, are well-suited for monitoring memory in limbic encephalitis [11, 18, 34, 45, 57].

While regression analyses were performed using raw scores, individual impairments were calculated using categorized sum scores across the different visual and verbal memory subfunctions (mean value of standardized memory parameters = 100 ± 10). On a categorical level, we rated visual or verbal memory performance as impaired if the memory score was more than a one-fold standard deviation below the standardized mean value, i.e., < 90.

Executive function was assessed via the second edition of the EpiTrack [31], which merges six subtests into one total score representing attention, executive function, and working memory; its clinical utility has been proven in monitoring drug treatment [55] and in screening new-onset epilepsies [56, 58]. Here, just as with memory, patients were rated as impaired if they scored below the mean minus one standard deviation of the healthy normative sample.

Mood was assessed via the Beck Depression Inventory (BDI) I [3]. Values > 10 were rated as an indicator for depressed mood.

As already mentioned in the section describing subject selection, the timing for evaluating patients with LE is crucial because the longitudinal follow-ups show, in part, considerable fluctuations in cognition over time [15]. It was therefore essential to synchronize neuropsychology, imaging, and the flow cytometry evaluations. Furthermore, since autoimmune treatments may well have negative effects on cognition, it is important to note that neuropsychological assessments, as a rule, were never carried out if the patient was being treated for an autoimmune condition.

### Statistical analyses

All statistics were performed using the IBM Statistical Package for the Social Sciences (SPSS) software, version 23. Descriptive statistics were performed to compare the three groups with regard to demographic, clinical–epileptological, neuropsychological, and flow cytometry data.

Categorical data were calculated via cross-tabulation statistics. The one-way analysis of variance (ANOVA) test was used to evaluate subgroup differences in continuous variables. The level of significance was set at *p* < 0.05.

Hierarchical, multiple stepwise regression analysis was applied separately for both groups to discern prediction models for cognition. The following hierarchical levels were considered as independent predictor variables in the following order of appearance: (1) demographic data (age, gender, education); (2) epilepsy-related data (age at onset, duration, number of antiepileptic drugs, individual drugs which are known to negatively impact cognition or mood, antidepressants); (3) structural MRI pathology findings (see above); and (4) results from flow cytometry analyses. The inclusion criterion for predictor variables was *p* < 0.05; the exclusion criterion was *p* > 0.1.

## Results

### LE patient subgroups

Antibody screening targets were GAD65, onconeural auto-antibodies with reported positive cases in the context of LE [1, 28, 29, 33, 36–38, 46], and neuronal surface membrane auto-antibodies. In total, 59 patients (56%) had no antibodies present in their CSF and PB, and were thus classified as antibody negative (AB−); 41 patients (38%) showed auto-antibodies against intracellular antigens (IAB+). Among the IAB+ patients, 17 (42%) were positive for GAD65, and 24 patients (58%) were positive for onconeural antibodies. It should be noted that the tumor rate is surprisingly low in onconeural antibody-positive patients, who are diagnosed in recent-onset unexplained epilepsies (3.2% in 93 patients) [18]. None of the patients included in this study had a tumor when screened with PET/CT, nor did any of the patients undergo anti-tumor treatment. The auto-antibodies with intracellular targets included those against the intracellular antigens Amphiphysin, CV2, GAD65, Ma2/Ta, Ri (ANNA2), SOX1, Yo (PCA1), and Zic4. Auto-antibodies against membrane surface antigens and synaptic proteins were identified in only six patients (6%) (SAB+). SAB+ included two patients with anti-LGI1, two patients with CASPR2, and two patients with anti-NMDAR autoantibodies.

Given the few number of patients with auto-antibodies against membrane surface antigens and synaptic proteins (*n* = 6), and because a relationship between antibody-load and cognition had already been demonstrated for this group, we excluded the SAB+ group from the subsequent analyses and focused only on IAB+ and AB− patients.

Using the ‘Graus criteria’ for possible and definite autoimmune LE, the clinical features of the IAB+ group fulfilled the criteria for a definitive LE diagnosis. However, the cognitive impairments often extended beyond working memory, and MRI pathology was often unilateral. The AB− group remained in the possible LE category [14].

### Demographics and clinical patient characteristics

This study finally comprised 100 patients, 55% male and 45% female, with an average age of 41 (± 15) years. A majority (72%) had an education of ≥10 years. Their age of epilepsy onset was late with an average age of 34 years (± 16 years) and the average duration of the epilepsy of 7 years (± 8 years). Most patients (84%) were taking antiepileptic drugs (AEDs) at the time of evaluation; 54% of them were on polytherapy; and approximately 9% were taking antidepressants. MRI amygdala pathologies (43%) were not observed signficantly more often than hippocampal pathologies (about 37%), but 84% of the amygdala pathologies were classified as swelling, and only 16% were classified as atrophy or signal intensity. In contrast, the pathology of the hippocampus was more often an atrophy or signal intensity (59%) rather than swelling (41%). Not a single patient was seizure free. The average monthly seizure frequency was 13 with a considerable variance (SD 58). Overall, 42% of the patients displayed a depressed mood.

As Table 1 illustrates, the two study groups’ demographic and clinical characteristics were largely comparable. The IAB+ patients tended to be older and had later onset epilepsies when compared to the AB− patients.

**Table 1:** Summary of demographic and clinical characteristics of LE patients

Parameters	Groups	IAB+ (*n* = 41)	AB− (*n* = 59)	*F*/*χ*^2^
Age (years)	M/SD	44.6 ± 16.5	38.9 ± 13.8	3.488 (*)
Sex (F)	*N* (%)	20 (48.8)	25 (42.4)	0.547 n.s.
Education (≥ 10 years)	*N* (%)	31 (75.0)	41 (69.5)	0.449 n.s.
IQ	M/SD	110.5 (15.4)	106.0 (12.3)	2.027 n.s.
Age at epilepsy onset (years)	M/SD	37.9 ± 17.6	31.6 ± 15.1	3.686 (*)
Duration of epilepsy (years)	M/SD	6.6 ± 9.2	7.2 ± 7.7	0.136 n.s.
Number of AEDs	M/SD	1.5 ± 0.9	1.6 ± 1.0	0.542 n.s
Off drug	*N* (%)	6 (14.6)	10 (16.9)	0.096 n.s.
Monotherapy	*N* (%)	14 (34.1)	16 (27.1)	0.569 n.s.
Polytherapy	*N* (%)	21 (51.2)	33 (55.9)	0.216 n.s
Antidepressants	*N* (%)	5 (12.2%)	4 (6.8%)	1.785 n.s
Neuroleptics	*N* (%)	–	–	–
Number of any Psychoactive drugs	M/SD	1.6 ± 0.9	1.7 ± 1.1	0.105 n.s
*Structural* *MRI*				
Side	*N* (%)			2.664 n.s
Right		9 (33)	13 (34)	
Left		7 (26)	16 (42)	
Bilateral		11 (41)	9 (24)	
Amygdala affected	*N* (%)	21 (51)	22 (34)	1.915 n.s
Swelling		17 (81)	19 (86)	
Atrophy		3 (14)	3 (14)	
Hyperintensity		1 (5)	0 (0)	
Hippocampus affected	*N* (%)	17 (51)	20 (37)	0.594 n.s
Swelling		8 (47)	7 (35)	
Atrophy		8 (47)	10 (50)	
Hyperintensity		1 (6)	3 (15)	
Seizure frequency	M/SD	9.2 ± 20.2	18.7 ± 58.7	0.600 n.s

*L* left, *R* right, *M* mean, *SD* standard deviation, *n* = number, *IAB+* intracellular auto-antibody positive, *AB−* auto-antibodies negative, *n.s*. not significant

(*)*p* < 0.1, * *p* < 0.05 (ANOVA or frequency tabulation with Chi^2^ test)

### Flow cytometry analysis

The absolute amounts of HLADR+  CD4+ T-cells, HLADR+  CD8+ T-cells, and CD19+ B-cells in cells per milliliter (cells/ml) are reported, as well as the percentage of the circulating lymphocytes in both CSF and PB using flow cytometry (see Table 2). With the exception of the IAB+ group’s higher percentage of HLADR+ CD4 and CD8 T-cells in the PB, and a tendency of more HLADR+ CD4 in CSF, the results did not differ significantly between the patient groups. HLADR+  CD4+ T-cell, HLADR+  CD8+ T-cell, and CD19+ B-cell concentrations (cells/ml) in blood and CSF were moderately correlated with each other with correlation coefficients ranging between 0.42 (*p* < 0.001) and 0.58 (*p* < 0.001).

**Table 2:** Flow cytometry results the different subgroups of LE patients

Parameters	Groups	IAB+ (*n* = 41)	AB− (*n* = 59)	*F*
HLADR+ CD4+ T cells CSF (cells/ml)	M/SD	105.8 ± 232.7	90.8 ± 153.6	0.150 n.s
HLADR+ CD4+ T cells CSF %^#^	M/SD	0.19 ± 0.13	0.16 ± 0.1	3.059 (*)
HLADR+ CD4+ T cells PB (cells/ml)	M/SD	50,647 ± 111,55	48,073 ± 117,81	0.931 n.s
HLADR+ CD4+ T cells PB %^#^	M/SD	0.09 ± 0.06	0.06 ± 0.05	9.858 **
HLADR+ CD8+ T cells CSF (cells/ml)	M/SD	47.1 ± 81.2	57.8 ± 70.4	0.484 n.s
HLADR+ CD8+ T cells CSF %^#^	M/SD	0.53 ± 0.18	0.51 ± 0.19	0.266 n.s
HLADR+ CD8+ T cells PB (cells/ml)	M/SD	32,77 ± 38,25	42,49 ± 86,80	0.442 n.s
HLADR+ CD8+ T cells PB %^#^	M/SD	0.25 ± 0.20	0.16 ± 0.15	6.782 *
CD19+ B Cells CSF (cells/ml)	M/SD	40.8 ± 214.9	6.04 ± 9.66	1.541 n.s
CD19+ B Cells PB (cells/ml)	M/SD	140,54 ± 282,59	207.29 ± 458,00	0.674 n.s

*M* mean, *SD* standard deviation, *n* number, *IAB+* intracellular auto-antibody positive, *AB−* auto-antibodies negative

^#^Corresponds to the following formula: *x*% = absolute numbers of HLADR+  CD4+ T-cells or HLADR+  CD8+ T-cells in PB or CSF (cells/ml) divided by absolute number CD4+ T-cells or CD8+ T-cells in PB or CSF (cells/ml)

*n.s.* not significant; (*)*p* < 0.1, *p < 0.05, **p < 0.01 ANOVA (F statistics) with post hoc analysis (LSD) or frequency tabulation with Chi^2^ test

### Cognitive performance and mood in LE patient subgroups

Analyses of the cognitive impairments in the patient subgroups revealed that between 34% (IAB+) and 40% (AB−) of the patients in the study groups displayed impaired verbal memory (total score), and between 43% (AB−) and 53% (IAB+) displayed impairments in visual memory (total score). Executive function was found to be impaired for 32% of both groups, mood was impaired for 46% of the patients in IAB+ , and for 42% in AB−. No significant group differences were observed on the level of the performance categories (% impaired). The groups did as well not differ when inference statistics were calculated (ANOVA results not specifically reported). Neither memory nor executive function or mood showed a relation to the monthly seizure frequency for the total group and individual subgroups (results not specifically reported).

**Fig. 1:**
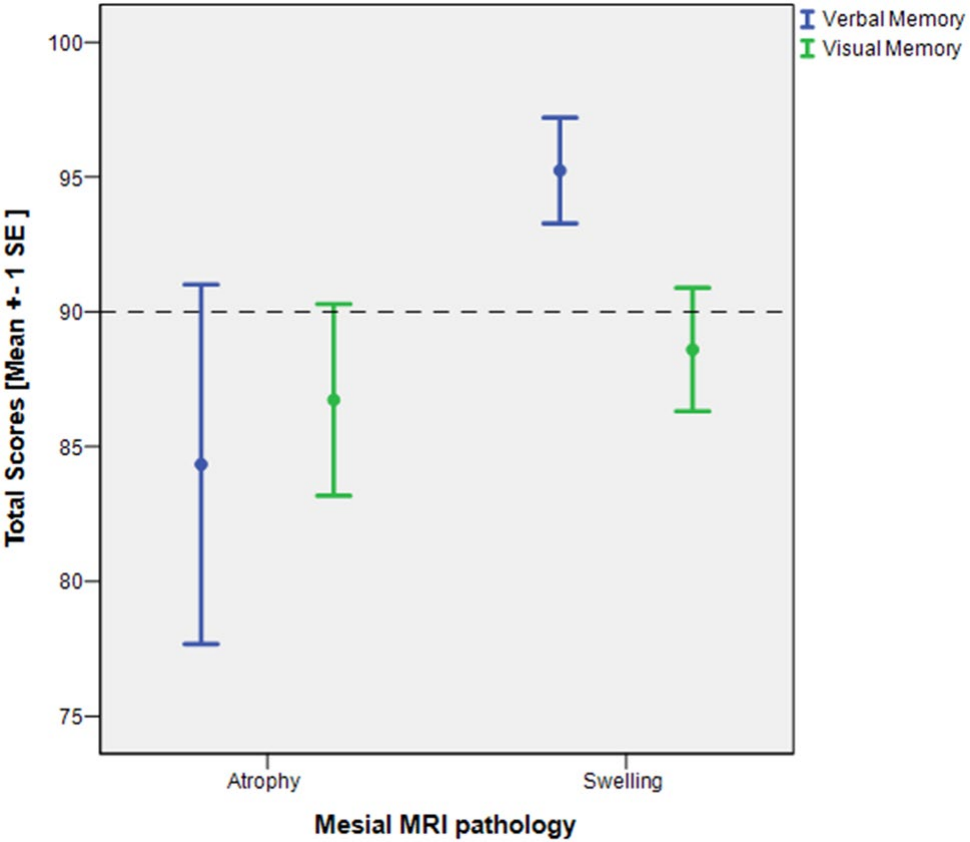
Verbal and visual memory as a function of MRI pathology. Total scores of verbal and visual memory are reported as standard values (100 ± 10). The scattered line indicates the threshold below which performances are classified as being within the impairment range

When evaluating the performances as a function of the lateralization of the pathology (left/right/bilateral), the total group also failed to yield any significant difference. However, an important finding is that the verbal memory performance (more so than visual memory) differed depending on the MRI pathology. Verbal memory (but not visual memory) was significantly worse in the presence of a temporo-mesial atrophy (volume decrease) as compared to a volume increase (swelling) of temporo-mesial structures (*F* = 4.9 *p* < 0.05 verbal memory, *F* = 0.177 *p* < 0.10 for visual memory) (see Fig. 1). Unlike verbal memory, visual memory was in the impairment range (standard value < 90) with either type of pathology. As stated above, the swelling (i.e., volume increase) was diagnosed more than twice as often than atrophy in the IAB+ (45% vs. 18%) and AB− groups (37% vs. 15%). This finding is also partly reflected in the regression analyses which differentiated further as to whether or not certain pathologies were seen left or right. Accordingly, bilateral hippocampal pathology and left hippocampal atrophy had an impact on cognitive performance in verbal memory (recognition) and executive function in the IAB+ group (see Table 3).

**Table 3:** Hierarchical regression models to predict cognition and mood in LE patients positive for intracellular auto-antibodies in CSF and PB. (IAB+)

Cognition and mood	Predictors	Beta weights	*T/*significance
*Verbal* *learning* *and* *memory* *(VLMT)*			
Learning model: *F* = 5.469, *p* < 0.05, *R*^2^ = 0.163	HLADR+ CD4+ T-cells in CSF %	− 0.404	− 2.339, *p* < 0.05
Memory (delayed recall) model: *F* = 5.967, *p* < 0.05, *R*^2^ = 0.176	HLADR+ CD4+ T-cells in CSF %	− 0.419	− 2.443, *p* < 0.05
Memory loss (after delay)	–	–	–
Recognition model: *F* = 11.715, *p* < 0.001, *R*^2^ = 0.652	Bilateral hippocampal pathology	− 0.518	− 4.194, *p* < 0.001
	HLADR+ CD4+ T-cells in CSF %	− 0.554	− 3.612, *p* < 0.01
	HLADR+ CD8+ T-cells in CSF %	− 0.424	− 2.794, *p* < 0.05
*Figural* *learning* *and* *memory* *(DCS-R)*			
Learning model: *F* = 4.993, *p* = 0.034, *R*^2^ = 0.156	Psychoactive drugs (number)	− 0.395	− 2.235, *p* < 0.05
Learning capacity model: *F* = 4.420, *p* = 0.045, *R*^2^ = 0.136	Education	0.369	2.102, *p* < 0.05
Recognition	–	–	–
*Executive* *functions* *(EpiTrack)*			
Model: *F* = 12.425, *p* < 0.001, *R*^2^ = 0.798	Education	0.589	5.492, *p* < 0.001
	Clobazam	− 0.466	− 4.742, *p* < 0.001
	Left hippocampal atrophy	−0.520	4.137, *p* < 0.001
	Age at onset	0.349	3.407, *p* < 0.01
	Bilateral hippocampal pathology	− 0.364	− 2.986, *p* < 0.01
	Swollen amygdala (right)	−0.236	2.130, *p* < 0.05
Mood (BDI I)			
Model: *F* = 4.25, *p* < 0.05, *R*^2^ = 0.111	Bilateral amygdala pathology	0.333	2.062, *p* < 0.05

### Association between immune cell loads and neuropsychological performance

Tables 3 and 4 summarize the hierarchical regression models to predict cognitive functions and mood based on the assumed predictors in LE patients in the two subgroups.

**Table 4:** Hierarchical regression models to predict cognitive performance in auto-antibody negative LE susceptive patients (AB−)

Cognitive functions	Predictors	*β* weights	*T*/significance
*Verbal* *learning* *and* *memory* *(VLMT)*			
Learning model: *F* = 15.238, *p* < 0.001, *R*^2^ = 0.498	Education	0.528	5.010, *p* < 0.001
	Female sex	0.333	3.184, *p* < 0.01
Memory (delayed recall) model: *F* = 18.668, *p* < 0.001, *R*^2^ = 0.443	Education	0.560	5.138, *p* < 0.001
	Female sex	0.370	3.395, *p* < 0.01
Memory loss (after delay) model: *F* = 7.968, *p* < 0.01, *R*^2^ = 0.142	Education	0.377	2.823, *p* < 0.01
Recognition model: *F* = 12.830, *p* < 0.001, *R*^2^ = 0.456	Education	0.523	4.801, *p* < 0.001
	Duration	0.279	2.493, *p* < 0.05
	Sex female	0.255	2.272, *p* < 0.05
*Figural* *learning* *and* *memory* *(DCS-R)*			
Learning model: *F* = 14.039, *p* < 0.001, *R*^2^ = 0.384	Education	0.567	4.828, *p* < 0.001
	CD19+ B-cells in CSF	− 0.303	− 2.792, *p* < 0.05
Learning capacity model: *F* = 14.554, *p* < 0.001, *R*^2^ = 0.388	Education	0.569	4.909, *p* < 0.001
	CD19+ B-cells in CSF	− 0.315	− 2.713, *p* < 0.01
Recognition model: *F* = 8.281, *p* = 0.001, *R*^2^ = 0.265	Education	0.420	3.316, *p* < 0.01
*Executive* *functions* *(EpiTrack)*			
Model: *F* = 6.824, *p* < 0.001, *R*^2^ = 0.367	Education	0.436	3.555, *p* < 0.01
	HLADR+ CD4+ T-cells in CSF %	− 0.327	− 2.781, *p* < 0.01
	HLADR+ CD4+ T-cells in PB (cells/ml)	− 0.284	− 2.340, *p* < 0.05
	Age at onset	0.219	1.759, *p* < 0.1
*Mood* *(BDI* *I)*			
Model: *F* = 8.21, *p* < 0.001, *R*^2^ = 0.286	Zonisamide	0.368	3.155, *p* < 0.01
	Duration of epilepsy	0.370	2.995, *p* < 0.01

**Fig. 2:**
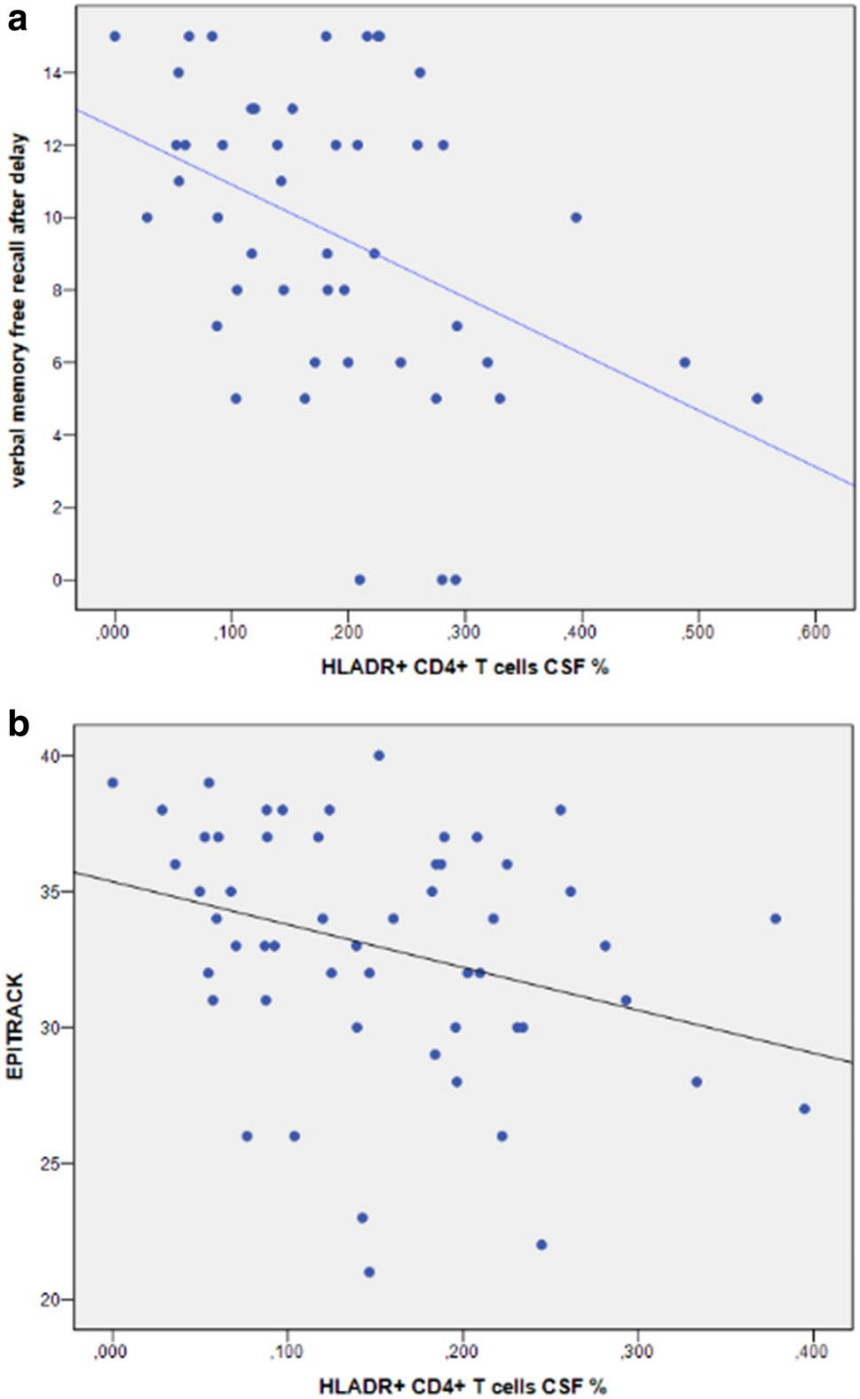
Immune cell loads and neuropsychological performance. **a** Correlations between the percentage of HLADR+  CD4+ T-cell in CSF and verbal memory (free recalled words after delay) in LE patients with intracellular auto-antibodies (*n* = 41). **b** Correlations between the percentage of HLADR+  CD4+ T-cell in CSF and executive function (EpiTrack) of autoantibody negative LE patients (*n* = 59). [The percentage of CD4+ T-cells in CSF was calculated according to the following formula: *x*% = absolute numbers of HLADR+  CD4+ T-cells in CSF (cells/ml) divided by absolute number CD4+ T-cells in CSF (cells/ml)]

In the IAB+ subgroup, the percentage of circulating HLADR+  CD4+ T-cells in CSF was inversely correlated with verbal recognition (*B* = − 0.554, *p* < 0.05), verbal delayed free recall (*B* = − 0.419, *p* < 0.05), and verbal learning performance (*B* = − 0.404, *p* < 0.05), respectively. The percentage of circulating HLADR+  CD8+ T-cells in CSF negatively correlated with verbal recognition (*B* = − 0.424, *p* = 0.01) (see Fig. 2a)

The AB− patient subgroup´s regression showed that the absolute amount of CD19+ B-cells in CSF was negatively correlated with visual learning (*B* = − 0.303, *p* < 0.05) and visual learning capacity (*B* = − 0.315, *p* < 0.05). In this subgroup, the EpiTrack score representing attention and executive function correlated negatively with the percentage of circulating HLADR+  CD4+ T-cells in CSF (*B* = − 0.327, *p* < 0.05) and the absolute HLADR+  CD4+ T-cell loads in PB (*B* = − 0.284, *p* < 0.05) (see Fig. 2b) Visual memory functions did not correlate with either the absolute amount or the percentage of the circulating of HLADR+  CD4+ T-cells and HLADR+  CD8+ T-cells in either CSF or PB as well as the absolute CD19+ B-cells loads in PB (*p* > 0.05). Finally we noted a relation between a longer duration of epilepsy, treatment with zonisamide (ZNS) and a negative mood in this group (*p* < 0.01).

Regression analysis revealed no correlation between the cognitive functions and structural abnormality in the AB− patients. In those patients with auto-antibodies against intracellular antigens (IAB+) in both CSF and PB, bilateral hippocampal pathology was found to correlate negatively with verbal recognition (*B* = − 0.518, *p* < 0.001) and executive function (*B* = − 0.364, *p* < 0.001). Attention and executive functions also correlated with left hippocampus atrophy (*B* = −0.520, *p* < 0.001) and right amygdala hypertrophy (*B* = −0.236, *p* < 0.05).

As expected, the factors education (better performance with better education), gender (women displayed better verbal memory performance and worse mood), and drugs (poorer memory performance with higher drug load, poorer performance in executive function particularly with clobazam, poorer mood with zonisamide) revealed some effects on the dependent measures apart from clinical and immunological factors (see results of regression analyses in Tables 3, 4).

## Discussion

The major objective of this study was to understand the relationship between objectively assessed cognitive impairments and subjective mood and immune cell loads of B- and T-cells among other clinical factors in larger groups of patients suffering from late-onset epilepsies with a definite LE diagnosis and proof of specific auto-antibodies, versus a group of patients with suspected LE and no evidence for auto-antibodies. A group with membrane cell-surface antibodies (SAB+) was excluded from analysis due to the small sample size. Hence, our evaluation focused on those patients with intracellular-positive antibodies (GAD 65 and paraneoplastic auto-antibodies) (IAB+), as compared to patients who were suspected to suffer from LE, but who were auto-antibody-negative (AB−).

These groups were indeed of major interest with regard to the question as to what determines cognitive and behavioral problems in LE. As for seizures, a highly relevant question with regard to cognition is the degree by which the symptoms are driven by and dependent upon the process of AE [4, 12, 42].

As mentioned in our introduction, there is some evidence of a relationship between hippocampal pathology and antibody load in patients with LE who are positive for VGKC (mainly LGI1) or NMDAR. Our study’s specific question was whether T-cell and B-cell autoimmune activity, as revealed by flow cytometry analyses, can serve as a biomarker for neuropsychological deficits in groups with antibodies against intra-cell antigens or suspected LE for which the MRI findings and antibody diagnostics cannot adequately explain cognitive and behavioral alterations.

After checking the demographic and clinical epileptological variables which are known to impair cognition in patients with epilepsy, the present data provide evidence that higher CD4+ T-cells load in CSF and, to a lesser degree, HLADR+  CD8+ T-cells in CSF, are linked to deficits in verbal memory parameters (learning, retrieval, recognition) in the intracellular auto-antibody positive group (IAB+), which coincide with the significantly higher CD4+ T-cells’ titers. Our antibody-negative subgroup (AB−) exhibited a significant correlation between the absolute amount of CD19+ B-cells in CSF and visual memory and learning capacity. Moreover, their CD4+ T-cell in PB and CF were associated with poorer executive function. As executive function is known to reflect frontal lobe function, frontal involvement, as indicated in GAD65 and VGKC-positive encephalitis, seems reasonable [11].

According to the literature, enlarged amygdala and/or hippocampal volumes occur frequently and are distinctive observations in LE patients [49]. In the IAB+ group, such enlargement was much more commonly found than atrophy (42% vs. 17%). A similar disparity was evident in the AB-group (34% vs. 14%). However, one must keep in mind that increased temporo-mesial volumes are an LE marker, thus the finding appears circular. Frequently, swelling in LE patients is initially observed over the course of the disease which indicates the acute phase which is then later followed by normalization under successful treatment or atrophy and sclerosis in case of disease progression. Assuming that this cross-sectional study cuts at different time points throughout the course of LE, the present findings may well reflect the pathological status under very different disease conditions.

Impairments in the evaluated cognitive domains and mood were comparable in two patient groups. With regard to the impact of structural MRI pathology, the present data reveal some interesting clues for understanding episodic memory impairment in LE. Those familiar with chronic temporal lobe epilepsies and memory are aware of the bias toward greater verbal or visual memory impairment, depending on whether the left language-dominant or right non-dominant hemisphere is affected [20], and also the sometimes strong correlation between impairments and the degree of cell loss in hippocampal subfields [52, 53]. In the present cohort, a lateralization effect was only partially evident, and hippocampal pathology (bilateral in IAB+) (rather than amygdala pathology), and atrophy (rather than the increased volume) is associated with memory impairment. Accordingly, one could speculate that the classic memory impairment known from chronic temporomesial epilepsy becomes evident in LE when the pathology migrates into the hippocampus and when, during later LE progression, the hippocampus becomes damaged in the form of atrophy and hippocampal sclerosis [11, 35]. This would be consistent with the observation that that accelerated long-term forgetting is often observed in the early phases of AE, whereas later with hippocampal sclerosis forgetfulness is observed already after a standard half hour retention interval [23].

Interestingly, our findings indicate that a poorer mood in the IAB+ group was related to bilateral amygdala pathology. Although this may point towards the amygdala’s potential role in depression and emotional liability in LE patients, it definitely requires deeper investigation [10]. Meanwhile, there is ample evidence suggesting that LE disrupts emotion regulation networks [2, 41].

Apart from the MRI pathologies and flow cytometry results in the regression models, our findings revealed additional evidence of the expected negative influence that a poorer education, an earlier onset and longer duration of epilepsy, and drug treatment have on cognition [7].

An important question which cannot be answered with the present data concerns the AB− group’s epilepsy etiology. This group, like the antibody positive group, was thought to suffer from LE because they fulfilled the clinical criteria for the diagnosis for LE. We cannot rule out that new, pathogenetically relevant antibodies in such patients will be detected in the future since novel antibody characterization paradigms have been recently established [39, 40]. Apart from the antibody finding, different T-cell loads in flow cytometry analysis, and a difference in chronological age and age at disease onset (both later in IAB+), the groups hardly differed in their clinical features. As previously noted, this can be expected due to the patient selection criteria. Without normative reference data on flow cytometry results, no criterion exists indicating which point within the results indicates a pathological status. Furthermore, since this was a cross-sectional study, eventual disease dynamics in epilepsy, MRI, neuropsychology, or behavior along with antiseizure and/or immunological treatment cannot contribute to a better understanding of this clinical group. A previous study in AB− patients, along with immunological treatment, revealed clinical changes and responses which, in part and in some patients, suggested an autoimmune etiology, despite the negative antibody findings [45].

### Limitations

The following shortcomings of the present study should be mentioned. First, some findings are circular since significant predictors reflect our patient selection criteria. However, in this regard, they confirm our selection criterion. Second, our cohort is heterogeneous and the LE subgroups still comprise low patient numbers and thus limit our findings’ statistical power. Third, as this was a cross-sectional study, patients were screened at presumably very different time points during their disease course. Even when the assessments are synchronized and the patient is not under the influence of any autoimmune treatment, evaluations which are conducted only once reveal just one point in time within the suggested but unknown course of the underlying disease. The occurrence of seizures does not mark the onset of. Neuropsychiatric and neuropsychological symptoms often precede seizure onset [54, 57]. Longitudinal assessments would be ideal for discerning which disease parameters enable us to monitor this disease and assess its inflammatory acuity, and eventually, treatment success. Fourth, many other factors determine the cognitive performance and behavior in patients with epilepsy and must also be taken into consideration. It is evident (also within this evaluation) that epilepsy originating from suspected LE, differs from what has been evaluated in recent decades within the context of epilepsy surgery in chronic mesio-temporal TLE. We attempted to take this into consideration via hierarchical regression analysis which successively factored in demographic, clinical data, treatment, and finally flow cytometry results. Our results reveal several definitive signals, while also substantiating this issue’s enormous complexity. Fifth, in the present study, we focused on the subsets of T- and B-cell populations which previously appeared to have a relevant impact in limbic encephalitis [13]. In future studies, it would be worthwhile to investigate further stratified subsets of cells including “activated” B cells, in addition to all T-cell populations. Finally, it should be noted that without control data as a reference point, the results from the flow cytometry analyses are difficult to interpret with regard to the pathological status. This may in part relativize the understanding of the absolute values and the meaningfulness of some correlations as well.

## Conclusion

This study confirms the association between memory impairment in patients with epilepsy and suspected LE and autoimmunological cellular mechanisms. A higher percentage of T-cells affected the memory performance of suspected LE patients with auto-antibodies targeting intracellular target structures negatively, whereas a higher amount of B-cells correlated with visual memory performance in auto-antibody negative LE patients. Future investigations will determine whether such disease parameters can serve to monitor disease dynamics and explain the neuropsychological courses for LE patient subgroups.

## References

[1] Adam VN, Budincevic H, Mrsic V, Stojcic EG, Matolic M, Markic A (2013). Paraneoplastic limbic encephalitis in a patient with adenocarcinoma of the colon: a case report. J Clin Anesth.

[2] Argyropoulos GPD, Moore L, Loane C, Roca-Fernandez A, Lage-Martinez C, Gurau O, Irani SR, Zeman A, Butler CR (2020). Pathologic tearfulness after limbic encephalitis: a novel disorder and its neural basis. Neurology.

[3] Beck A, Steer R (1987). Beck depression inventory manual.

[4] Britton JW, Dalmau J (2019). Recognizing autoimmune encephalitis as a cause of seizures: treating cause and not effect. Neurology.

[5] Butler CR, Miller TD, Kaur MS, Baker IW, Boothroyd GD, Illman NA, Rosenthal CR, Vincent A, Buckley CJ (2014). Persistent anterograde amnesia following limbic encephalitis associated with antibodies to the voltage-gated potassium channel complex. J Neurol Neurosurg Psychiatry.

[6] Doelken MT, Mennecke A, Huppertz HJ, Rampp S, Lukacs E, Kasper BS, Kuwert T, Ritt P, Doerfler A, Stefan H, Hammen T (2012). Multimodality approach in cryptogenic epilepsy with focus on morphometric 3T MRI. J Neuroradiol.

[7] Elger CE, Helmstaedter C, Kurthen M (2004). Chronic epilepsy and cognition. Lancet Neurol.

[8] Finelli PF (2011). Autoimmune limbic encephalitis with GAD antibodies. Neurohospitalist.

[9] Finke C, Kopp UA, Pajkert A, Behrens JR, Leypoldt F, Wuerfel JT, Ploner CJ, Pruss H, Paul F (2016). Structural hippocampal damage following anti-*N*-methyl-d-aspartate receptor encephalitis. Biol Psychiatry.

[10] Frisch C, Hanke J, Kleineruschkamp S, Roske S, Kaaden S, Elger CE, Schramm J, Yilmazer-Hanke DM, Helmstaedter C (2009). Positive correlation between the density of neuropeptide y positive neurons in the amygdala and parameters of self-reported anxiety and depression in mesiotemporal lobe epilepsy patients. Biol Psychiatry.

[11] Frisch C, Malter MP, Elger CE, Helmstaedter C (2013). Neuropsychological course of voltage-gated potassium channel and glutamic acid decarboxylase antibody related limbic encephalitis. Eur J Neurol.

[12] Geis C, Planaguma J, Carreno M, Graus F, Dalmau J (2019). Autoimmune seizures and epilepsy. J Clin Invest.

[13] Golombeck KS, Bonte K, Monig C, van Loo KM, Hartwig M, Schwindt W, Widman G, Lindenau M, Becker AJ, Glatzel M, Elger CE, Wiendl H, Meuth SG, Lohmann H, Gross CC, Melzer N (2016). Evidence of a pathogenic role for CD8(+) T cells in anti-GABAB receptor limbic encephalitis. Neurol Neuroimmunol Neuroinflamm.

[14] Graus F, Titulaer MJ, Balu R, Benseler S, Bien CG, Cellucci T, Cortese I, Dale RC, Gelfand JM, Geschwind M, Glaser CA, Honnorat J, Hoftberger R, Iizuka T, Irani SR, Lancaster E, Leypoldt F, Pruss H, Rae-Grant A, Reindl M, Rosenfeld MR, Rostasy K, Saiz A, Venkatesan A, Vincent A, Wandinger KP, Waters P, Dalmau J (2016). A clinical approach to diagnosis of autoimmune encephalitis. Lancet Neurol.

[15] Hansen N, Ernst L, Ruber T, Widman G, Becker AJ, Elger CE, Helmstaedter C (2018). Pre- and long-term postoperative courses of hippocampus-associated memory impairment in epilepsy patients with antibody-associated limbic encephalitis and selective amygdalohippocampectomy. Epilepsy Behav.

[16] Hansen N, Schwing K, Onder D, Widman G, Leelaarporn P, Prusseit I, Surges R, Melzer N, Gross C, Becker AJ, Witt JA, Elger CE, Helmstaedter C (2020). Low CSF CD4/CD8+ T-cell proportions are associated with blood-CSF barrier dysfunction in limbic encephalitis. Epilepsy Behav.

[17] Hansen N, Widman G, Schwing K, Leelaarporn P, Onder D, Prusseit I, Surges R, Becker AJ, Witt JA, Helmstaedter C, Elger CE (2020). CD19+B-cells contribute to auto-antibody-negative limbic encephalitis. Epilepsy Behav.

[18] Hansen N, Widman G, Stuff S, Becker AJ, Witt JA, Ahmadzadehfar H, Helmstaedter C, Elger CE (2018). Cancer frequency detected by positron emission tomography-computed tomography in limbic encephalitis. Epilepsy Behav.

[19] Hansen N, Widman G, Witt JA, Wagner J, Becker AJ, Elger CE, Helmstaedter C (2016). Seizure control and cognitive improvement via immunotherapy in late onset epilepsy patients with paraneoplastic versus GAD65 autoantibody-associated limbic encephalitis. Epilepsy Behav.

[20] Helmstaedter C (2002). Effects of chronic epilepsy on declarative memory systems. Prog Brain Res.

[21] Helmstaedter C, Lendt M, Lux S (2001). VLMT Verbaler Lern- und Merkfähigkeitstest.

[22] Helmstaedter C, Pohl C, Hufnagel A, Elger CE (1991). Visual learning deficits in nonresected patients with right temporal lobe epilepsy. Cortex.

[23] Helmstaedter C, Winter B, Melzer N, Lohmann H, Witt JA (2019). Accelerated long-term forgetting in focal epilepsies with special consideration given to patients with diagnosed and suspected limbic encephalitis. Cortex.

[24] Helmstaedter C, Witt JA (2012). Clinical neuropsychology in epilepsy: theoretical and practical issues. Handb Clin Neurol.

[25] Heming M, Lohmann L, Schulte-Mecklenbeck A, Brix T, Gross CC, Wiendl H, Klotz L, Meyer Z, Horste G (2020). Leukocyte profiles in blood and CSF distinguish neurosarcoidosis from multiple sclerosis. J Neuroimmunol.

[26] Heming M, Schulte-Mecklenbeck A, Brix T, Wolbert J, Ruland T, Klotz L, Meuth SG, Gross CC, Wiendl H, Meyer Z, Horste G (2019). Immune cell profiling of the cerebrospinal fluid provides pathogenetic insights into inflammatory neuropathies. Front Immunol.

[27] Hermetter C, Fazekas F, Hochmeister S (2018). Systematic review: syndromes, early diagnosis, and treatment in autoimmune encephalitis. Front Neurol.

[28] Kay L, Bauer S, Koczulla AR, Librizzi D, Vadasz D, Knake S, Rosenow F, Strzelczyk A (2018). Ondine's curse and temporal lobe seizures: anti-Hu- and Zic4-associated paraneoplastic brainstem and limbic encephalitis. Eur J Neurol.

[29] Kunstreich M, Kreth JH, Oommen PT, Schaper J, Karenfort M, Aktas O, Tibussek D, Distelmaier F, Borkhardt A, Kuhlen M (2017). Paraneoplastic limbic encephalitis with SOX1 and PCA2 antibodies and relapsing neurological symptoms in an adolescent with Hodgkin lymphoma. Eur J Paediatr Neurol.

[30] Lamberti G, Weidlich S (1999). DCS: a visual learning and memory test for neuropsychological assessment.

[31] Lutz MT, Helmstaedter C (2005). EpiTrack: tracking cognitive side effects of medication on attention and executive functions in patients with epilepsy. Epilepsy Behav.

[32] Lux S, Helmstaedter C, Elger CE (1999). Normative study on the "Verbaler Lern- und Merkfahigkeitstest" (VLMT). Diagnostica.

[33] Malter MP, Helmstaedter C, Urbach H, Vincent A, Bien CG (2010). Antibodies to glutamic acid decarboxylase define a form of limbic encephalitis. Ann Neurol.

[34] Malter MP, Widman G, Galldiks N, Stoecker W, Helmstaedter C, Elger CE, Wagner J (2016). Suspected new-onset autoimmune temporal lobe epilepsy with amygdala enlargement. Epilepsia.

[35] Miller TD, Chong TT, Aimola Davies AM, Ng TWC, Johnson MR, Irani SR, Vincent A, Husain M, Jacob S, Maddison P, Kennard C, Gowland PA, Rosenthal CR (2017). Focal CA3 hippocampal subfield atrophy following LGI1 VGKC-complex antibody limbic encephalitis. Brain.

[36] Mitchell AN, Bakhos CT, Zimmerman EA (2015). Anti-Ri-associated paraneoplastic brainstem cerebellar syndrome with coexisting limbic encephalitis in a patient with mixed large cell neuroendocrine lung carcinoma. J Clin Neurosci.

[37] Monstad SE, Nostbakken JK, Vedeler CA (2009). CRMP5 antibodies found in a patient with limbic encephalitis and myasthenia gravis. J Neurol Neurosurg Psychiatry.

[38] Ortega Suero G, Sola-Valls N, Escudero D, Saiz A, Graus F (2018). Anti-Ma and anti-Ma2-associated paraneoplastic neurological syndromes. Neurologia.

[39] Pitsch J, Kamalizade D, Braun A, Kuehn JC, Gulakova PE, Rueber T, Lubec G, Dietrich D, von Wrede R, Helmstaedter C, Surges R, Elger CE, Hattingen E, Vatter H, Schoch S, Becker AJ (2020). Drebrin autoantibodies in patients with seizures and suspected encephalitis. Ann Neurol.

[40] Scharf M, Miske R, Heidenreich F, Giess R, Landwehr P, Blocker IM, Begemann N, Denno Y, Tiede S, Dahnrich C, Schlumberger W, Unger M, Teegen B, Stocker W, Probst C, Komorowski L (2015). Neuronal Na+/K+ ATPase is an autoantibody target in paraneoplastic neurologic syndrome. Neurology.

[41] Schroder O, Schriewer E, Golombeck KS, Kurten J, Lohmann H, Schwindt W, Wiendl H, Bruchmann M, Melzer N, Straube T (2015). Impaired autonomic responses to emotional stimuli in autoimmune limbic encephalitis. Front Neurol.

[42] Steriade C, Britton J, Dale RC, Gadoth A, Irani SR, Linnoila J, McKeon A, Shao XQ, Venegas V, Bien CG (2020). Acute symptomatic seizures secondary to autoimmune encephalitis and autoimmune-associated epilepsy: conceptual definitions. Epilepsia.

[43] Tuzun E, Dalmau J (2007). Limbic encephalitis and variants: classification, diagnosis and treatment. Neurologist.

[44] Urbach H, Rauer S, Mader I, Paus S, Wagner J, Malter MP, Pruss H, Lewerenz J, Kassubek J, Hegen H, Auer M, Deisenhammer F, Ufer F, Bien CG, Baumgartner A (2015). Supratentorial white matter blurring associated with voltage-gated potassium channel-complex limbic encephalitis. Neuroradiology.

[45] von Rhein B, Wagner J, Widman G, Malter MP, Elger CE, Helmstaedter C (2017). Suspected antibody negative autoimmune limbic encephalitis: outcome of immunotherapy. Acta Neurol Scand.

[46] Wagner-Altendorf TA, Wandinger KP, Frydrychowicz A, Merseburger AS, Munte TF (2019). Anti-Amphiphysin-associated limbic encephalitis in a 72-year-old patient with aortic angiosarcoma. BMJ Case Rep.

[47] Wagner J, Schoene-Bake JC, Malter MP, Urbach H, Huppertz HJ, Elger CE, Weber B (2013). Quantitative FLAIR analysis indicates predominant affection of the amygdala in antibody-associated limbic encephalitis. Epilepsia.

[48] Wagner J, Schoene-Bake JC, Witt JA, Helmstaedter C, Malter MP, Stoecker W, Probst C, Weber B, Elger CE (2016). Distinct white matter integrity in glutamic acid decarboxylase and voltage-gated potassium channel-complex antibody-associated limbic encephalitis. Epilepsia.

[49] Wagner J, Witt JA, Helmstaedter C, Malter MP, Weber B, Elger CE (2015). Automated volumetry of the mesiotemporal structures in antibody-associated limbic encephalitis. J Neurol Neurosurg Psychiatry.

[50] Widman G, Golombeck K, Hautzel H, Gross CC, Quesada CM, Witt JA, Rota-Kops E, Ermert J, Greschus S, Surges R, Helmstaedter C, Wiendl H, Melzer N, Elger CE (2015). Treating a GAD65 antibody-associated limbic encephalitis with basiliximab: a case study. Front Neurol.

[51] Widman G, Schulte-Mecklenbeck A, Witt J, Dik A, Pitsch J, Golembeck KS, Wagner J, Moenig C, Strippel C, Johnen A, Kuhlmann T, Wiendl H, Elger CE, Meuth SG, Helmstaedter C, Gross C, Becker AJ, Melzer N (2018) CD8+ T cell-mediated epileptic neurodegeneration in limbic encephalitis associated with glutamic acid decarboxylase autoantibodies

[52] Witt JA, Coras R, Becker AJ, Elger CE, Blumcke I, Helmstaedter C (2019). When does conscious memory become dependent on the hippocampus? The role of memory load and the differential relevance of left hippocampal integrity for short- and long-term aspects of verbal memory performance. Brain Struct Funct.

[53] Witt JA, Coras R, Schramm J, Becker AJ, Elger CE, Blumcke I, Helmstaedter C (2014). The overall pathological status of the left hippocampus determines preoperative verbal memory performance in left mesial temporal lobe epilepsy. Hippocampus.

[54] Witt JA, Helmstaedter C (2017). Cognition in epilepsy: current clinical issues of interest. Curr Opin Neurol.

[55] Witt JA, Helmstaedter C (2013). Monitoring the cognitive effects of antiepileptic pharmacotherapy–approaching the individual patient. Epilepsy Behav.

[56] Witt JA, Helmstaedter C (2012). Should cognition be screened in new-onset epilepsies? A study in 247 untreated patients. J Neurol.

[57] Witt JA, Vogt VL, Widman G, Langen KJ, Elger CE, Helmstaedter C (2015). Loss of autonoetic awareness of recent autobiographical episodes and accelerated long-term forgetting in a patient with previously unrecognized glutamic acid decarboxylase antibody related limbic encephalitis. Front Neurol.

[58] Witt JA, Werhahn KJ, Kramer G, Ruckes C, Trinka E, Helmstaedter C (2014). Cognitive-behavioral screening in elderly patients with new-onset epilepsy before treatment. Acta Neurol Scand.

